# Practical calculation method to estimate the absolute boron concentration in tissues using ^18^F-FBPA PET

**DOI:** 10.1007/s12149-017-1172-5

**Published:** 2017-04-24

**Authors:** Tadashi Watabe, Kohei Hanaoka, Sadahiro Naka, Yasukazu Kanai, Hayato Ikeda, Masanao Aoki, Eku Shimosegawa, Mitsunori Kirihata, Jun Hatazawa

**Affiliations:** 1 Department of Nuclear Medicine and Tracer Kinetics, Osaka University Graduate School of Medicine, 2-2 Yamadaoka, Suita, Osaka 565-0871 Japan; 2 Medical Imaging Center for Translational Research, Osaka University Graduate School of Medicine, Suita, Osaka Japan; 3 Department of Molecular Imaging in Medicine, Osaka University Graduate School of Medicine, Suita, Osaka Japan; 4 Osaka University Hospital, Suita, Osaka Japan; 5 Osaka Prefectural University, Sakai, Osaka Japan; 6 Immunology Frontier Research Center, Osaka University, Suita, Osaka Japan

**Keywords:** FBPA, Positron emission tomography, Boron concentration

## Abstract

**Purpose:**

The purpose of this study was to establish a practical method to estimate the absolute boron concentrations in the tissues based on the standardized uptake values (SUVs) after administration of 4-borono-phenylalanine (BPA) using 4-borono-2-^18^F-fluoro-phenylalanine (^18^F-FBPA) PET.

**Methods:**

Rat xenograft models of C6 glioma (*n* = 7, body weight 241 ± 28.0 g) were used for the study. PET was performed 60 min after intravenous injection of ^18^F-FBPA (30.5 ± 0.7 MBq). After the PET scanning, BPA-fructose (167.3 ± 18.65 mg/kg) was administered by slow intravenous injection to the same subjects. The rats were killed 60 min after the BPA injection and tissue samples were collected from the major organs and tumors. The absolute boron concentrations (unit: ppm) in the samples were measured by inductively coupled plasma optical emission spectrometry (ICP-OES). The boron concentrations in the tissues/tumors were also estimated from the ^18^F-FBPA PET images using the following formula: estimated absolute boron concentration (ppm) = 0.0478 × [BPA dose (mg/kg)] × SUV. The measured absolute boron concentrations (mBC) by ICP-OES and the estimated boron concentrations (eBC) from the PET images were compared.

**Results:**

The percent difference between the mBC and eBC calculated based on the SUV_max_ was −5.2 ± 21.1% for the blood, −9.4 ± 22.3% for the brain, 1.6 ± 21.3% for the liver, −14.3 ± 16.8% for the spleen, −9.5 ± 27.5% for the pancreas, and 3.4 ± 43.2% for the tumor. Relatively large underestimation was observed for the lung (−48.4 ± 16.2%), small intestine (−37.8 ± 19.3%) and large intestine (−33.9 ± 11.0%), due to the partial volume effect arising from the air or feces contained in these organs. In contrast, relatively large overestimation was observed for the kidney (34.3 ± 29.3%), due to the influence of the high uptake in urine.

**Conclusions:**

The absolute boron concentrations in tissues/tumors can be estimated from the SUVs on ^18^F-FBPA PET using a practical formula. Caution must be exercised in interpreting the estimated boron concentrations in the lung, small intestine and large intestine, to prevent the adverse effects of overexposure, which could occur due to underestimation by partial volume effect using PET.

**Electronic supplementary material:**

The online version of this article (doi:10.1007/s12149-017-1172-5) contains supplementary material, which is available to authorized users.

## Introduction

Boron neutron capture therapy (BNCT) is an effective treatment method for recurrent glioma, malignant melanoma, and various other head and neck cancers [1–3]. 4-borono-phenylalanine (BPA) is used as the major carrier of boron-10 (^10^B) in BNCT. 4-borono-2-^18^F-fluoro-phenylalanine (^18^F-FBPA) PET is usually performed before BNCT and the accumulations in the tumor and normal tissues are evaluated by determining the relative uptake ratios [4]. If the tumor to normal tissue (T/N ratio) or tumor to blood (T/B ratio) is more than 2.5, BNCT is considered to be indicated [5]. However, to prevent the adverse effects of the alpha-particles emitted from ^10^B after neutron capture, estimation of the absolute boron concentrations in the normal tissues is essential. Our previous study reported the feasibility of estimating the ^10^B concentrations in normal organs based on ppm in ^18^F-FBPA PET before neutron irradiation in BNCT [6]. However, simplification of the calculation method and validation of the method of estimation of the ^10^B concentration from PET images are essential before it can be applied in routine clinical practice. The purpose of this study was to establish a practical method to estimate the absolute boron concentrations in tissues after administration of BPA based on the standardized uptake values (SUVs) determined from ^18^F-FBPA PET images as well as to validate the accuracy of the method.

## Methods

Male F344 rats (*n* = 7, body weight 241.7 ± 28.0 g, 11–13 weeks old) were purchased from Charles River Japan, Inc. (Atsugi, Japan). A rat glioma C6 cell line, derived from gliomas induced by *N*-nitrosomethylurea, was provided by the RIKEN BRC (Tsukuba, Japan). Rat xenograft models of C6 glioma were evaluated 3 weeks after the tumor implantation in the subcutaneous region of either side [7]. All animal experiments were performed in compliance with the guidelines of the Institute of Experimental Animal Sciences. The study protocol was approved by the Animal Care and Use Committee of the Osaka University Graduate School of Medicine (Approval Number: 20-144-008).

^18^F-FBPA was prepared as described previously, using an F-1 synthesizer (Sumitomo Heavy Industries, Tokyo, Japan) [7]. PET/CT data were acquired with a small-animal PET system (Inveon PET/CT system, Siemens Medical Solutions) [8]. PET scanning was performed 60 min after intravenous injection of ^18^F-FBPA (30.5 ± 0.7 MBq) into the animals under isoflurane anesthesia (2% plus oxygen). CT was performed before or after the PET acquisition. All PET data were reconstructed using 2-dimensional ordered-subset expectation maximization (16 subsets, 4 iterations), with attenuation and scatter correction. Regional uptake of radioactivity was decay-corrected for the injection time and expressed as the SUV corrected for the injected dose (MBq) and body weight (g). Volumes of interest (VOIs) were placed in the brain, lung, liver, spleen, pancreas, small intestine, large intestine, kidney, tumor, and blood pool in the left ventricle on the PET images with reference to the CT images, using the PMOD software (Ver. 3.404).

After the PET scanning, BPA-fructose (167.32 ± 18.65 mg/kg) was administered by slow intravenous injection over 2 min to the same subjects. Rats were killed 60 min after the BPA injection and tissue samples were collected from the major organs and tumors. The absolute boron concentrations (unit: ppm) in the samples were measured by inductively coupled plasma optical emission spectrometry (ICP-OES) [9].

Boron concentrations in the major organs/tumor were also estimated from the ^18^F-FBPA PET images using the following formula.

$${\text{Estimated boron concentration }}({\text{ppm}}) = 0.0478 \times [{\text{BPA dose }}({\text{mg/kg}})] \times {\text{SUV}} .$$

The factor of 0.0478 was derived from the molecular weight ratio of boron to BPA (calculated as 10/209.01). The BPA dose (mg/kg) was defined as the injected BPA dose (mg) per unit body weight (kg). SUV was calculated as radioactivity measured by PET (Bq/g) divided by injected radioactivity per body weight (Bq/g), where the specific gravity of the tissue was assumed as 1.0 (g/ml). The maximum SUV (SUV_max_) or mean SUV (SUV_mean_) calculated from the VOIs on the ^18^F-FBPA PET images was used for the calculation.

The measured absolute boron concentrations (mBC) by ICP-OES and the estimated boron concentrations (eBC) from the PET images in the major organs and the tumor were compared by a paired *t* test. The percent differences between the eBC and mBC were also evaluated and compared between SUV_max_-based estimation and SUV_mean_-based estimation. Statistical analyses were carried out using the SPSS, version 19.0 (SPSS, Chicago, IL, USA), and probability values of less than 0.05 were considered to denote statistical significance.

## Results

Comparisons between the mBC and eBC values are shown in Table 1. The eBC values calculated from the SUV_mean_ were significantly smaller than the mBC values for the blood, brain, lung, liver, spleen, pancreas, small intestine, large intestine, and the tumor. The eBC values calculated from the SUV_max_ were also significantly smaller than the mBC values for the lung, small intestine and large intestine, and significantly larger for the kidney. The percent differences between the mBC and eBC based on SUV_max_ calculation were −5.2 ± 21.1% for the blood, −9.4 ± 22.3% for the brain, 1.6 ± 21.3% for the liver, −14.3 ± 16.8% for the spleen, −9.5 ± 27.5% for the pancreas, and 3.4 ± 43.2% for the tumor. Relatively large underestimation was observed for the lung (−48.4 ± 16.2%), small intestine (−37.8 ± 19.3%) and large intestine (−33.9 ± 11.0%), possibly due to the partial volume effect arising from the air or feces contained in these organs. In contrast, large overestimation was observed for the kidney (34.3 ± 29.3%) due to the influence of the high uptake in urine.

**Table 1:** Comparison between measured and calculated boron concentrations (ppm) and percent difference between the mBC and eBC (%)

	mBC by ICP-OES (ppm)	eBC from SUV_max_ (ppm)	eBC from SUV_mean_ (ppm)	Difference between mBC and eBC from SUV_max_ (%)	Difference between mBC and eBC from SUV_mean_ (%)
Blood	12.2 ± 1.0	11.6 ± 2.7	9.0 ± 1.9**	−5.2 ± 21.1	−26.6 ± 12.6
Brain	7.6 ± 1.1	8.2 ± 1.3	5.3 ± 1.3**	9.4 ± 22.3	−30.1 ± 19.0
Lung	15.0 ± 3.9	7.3 ± 1.0**	4.9 ± 1.0**	−48.4 ± 16.2	−65.7 ± 12.1
Liver	14.9 ± 2.4	14.9 ± 2.6	11.6 ± 2.3*	1.6 ± 21.3	−20.8 ± 18.8
Spleen	20.4 ± 4.7	16.9 ± 2.0	11.0 ± 1.5**	−14.3 ± 16.8	−44.0 ± 13.3
Pancreas	52.2 ± 12.1	46.2 ± 12.1	31.2 ± 7.0**	−9.5 ± 27.5	−37.4 ± 23.6
Small intestine	16.4 ± 2.5	9.9 ± 1.9**	6.9 ± 1.5**	−37.8 ± 19.3	−56.7 ± 11.8
Large intestine	13.2 ± 1.5	8.7 ± 1.6**	5.8 ± 1.4**	−33.9 ± 11.0	−55.4 ± 11.8
Kidney	83.1 ± 16.1	108.9 ± 17.1*	89.1 ± 14.5	34.3 ± 29.3	10.0 ± 24.9
Tumor	27.6 ± 8.5	26.0 ± 6.0	15.2 ± 3.4**	3.4 ± 43.2	−42.4 ± 16.5

** *p* < 0.01 and * *p* < 0.05 by paired *t* test

## Discussion

Our previous study demonstrated the existence of a significant positive correlation between the accumulation levels of BPA and ^18^F-FBPA [9]. In the present study, we demonstrated the feasibility of estimating the absolute boron concentrations in tissues/tumors after administration of BPA based on the SUVs determined from ^18^F-FBPA PET images, which also showed good correlations between the boron concentration and the ^18^F-FBPA uptake, consistent with our previous report (Supplemental Fig. 1). Furthermore, we found that more accurate estimation was afforded by the SUV_max_ than by the SUV_mean_, except for the case of the kidney. SUV_max_ is a major, frequently used index in clinical oncology practice; therefore, the method described herein can easily be applied in routine clinical practice.

Estimation of the absolute boron concentrations in normal tissues is important, because alpha-particles from ^10^B show large energy transfer and have the potential to cause severe adverse effects in the event of overexposure of the neutron beam [10]. Careful estimation is essential, especially when BNCT is applied for lung or abdominal cancers. Relatively large underestimation was observed for the lung, small intestine and large intestine, due to the partial volume effect. Caution is needed, because underestimation of the tissue boron concentration might lead to excessive radiation exposure of the corresponding tissues. In contrast, large overestimation was observed for the kidney (34.3 ± 29.3%); the percent difference for the kidney was 10.0 ± 24.9% in the SUV_mean_-based estimation, which is considered to be optimal for the kidney. In the tumors, the relationship between mBC and eBC depends on the intratumoral heterogeneity of C6 glioma. Since we measured the whole tumor content for the measurement of boron concentration by ICP-OES, mBC showed smaller values with the increase of necrotic regions. Whereas, eBC from SUVmax reflected the hottest region of the tumor, which showed a certain degree of variability (Supplemental Fig. 2). As a result, standard deviation of the percent difference in the tumor (43.2%) was relatively large as compared to that for the major organs.

We recently reported about the intratumoral heterogeneity with hypoxic and necrotic regions in the C6 glioma xenograft by comparing ^18^F-FMISO and ^15^O-labeled gas PET to histological analysis [11]. Another previous study reported the characteristic difference in the tumor xenograft by comparing three tumor cell lines [12]. In that study, U251 (human glioblastoma) xenograft showed the necrotic region with the minimal stromal component, whereas BxPC-3 (human pancreatic ductal adenocarcinomas) showed abundant stroma and no apparent necrotic region. Tumor-associated stroma is the key determinant for the tumor hypoxia and necrosis which is related to the intratumoral heterogeneity.

Our previous study demonstrated the feasibility of estimating the tissue/tumor boron concentration using ^18^F-FBPA PET in humans [6]. In the present study, we simplified the estimation method using SUV and validated its accuracy by comparing the results with the actually measured boron concentrations. The estimated image map of boron concentration (unit: ppm) can be obtained from the PET images (unit: SUV) by simply multiplying with a coefficient, calculated as 0.0478 × [BPA dose (mg/kg)].

In this study, we used the molecular weight of BPA as a simple substance, not as the complexed compound. BPA complexed solutions, such as BPA-fructose or BPA-mannitol, are usually used for intravenous infusion because of the low solubility of BPA at physiological pH [13, 14]. If the molecular weight of BPA-fructose is applied, coefficient is calculated as 0.0259 × [BPA-fructose dose (mg/kg)] in the formula. However, fructose and mannitol are solubilizing agents, and there is room for further optimization. The infusion dose of BPA (mg/kg) should be normalized using the weight of the simple BPA compound for accurate comparisons among studies.

A limitation of our study is related to the administration method of BPA. We used slow bolus injection of BPA over 2 min, similar to that for ^18^F-FBPA injection. However, continuous infusion of BPA is often used in the clinical procedure of BNCT [15]. For practical use for clinical setting of BNCT, further study with comparing mBC by drip infusion and eBC might be needed.

## Conclusions

The absolute boron concentrations in tissues/tumors can be estimated from the SUVs determined by ^18^F-FBPA PET using a simple formula, suggesting the feasibility of this method in clinical practice. However, caution must be exercised in interpreting the estimated boron concentrations in the lung, small intestine and large intestine to prevent the adverse effects of neutron beam overexposure, which could arise as a result of underestimation by partial volume effect using PET.

## Electronic supplementary material

Below is the link to the electronic supplementary material.

Supplementary material 1 (DOC 1606 kb)

## References

[1] Nariai T, Ishiwata K, Kimura Y, Inaji M, Momose T, Yamamoto T (2009). PET pharmacokinetic analysis to estimate boron concentration in tumor and brain as a guide to plan BNCT for malignant cerebral glioma. Appl Radiat Isot.

[2] Ishiwata K, Ido T, Honda C, Kawamura M, Ichihashi M, Mishima Y (1992). 4-Borono-2-[^18^F]fluoro-d, l-phenylalanine: a possible tracer for melanoma diagnosis with PET. Int J Radiat Appl Instrum B.

[3] Kato I, Fujita Y, Maruhashi A, Kumada H, Ohmae M, Kirihata M (2009). Effectiveness of boron neutron capture therapy for recurrent head and neck malignancies. Appl Radiat Isot.

[4] Isohashi K, Shimosegawa E, Naka S, Kanai Y, Horitsugi G, Mochida I (2016). Comparison of the image-derived radioactivity and blood-sample radioactivity for estimating the clinical indicators of the efficacy of boron neutron capture therapy (BNCT): 4-borono-2-^18^F-fluoro-phenylalanine (FBPA) PET study. EJNMMI Res.

[5] Aihara T, Morita N, Kamitani N, Kumada H, Ono K, Hiratsuka J (2014). Boron neutron capture therapy for advanced salivary gland carcinoma in head and neck. Int J Clin Oncol.

[6] Shimosegawa E, Isohashi K, Naka S, Horitsugi G, Hatazawa J (2016). Assessment of 10B concentration in boron neutron capture therapy: potential of image-guided therapy using ^18^FBPA PET. Ann Nucl Med.

[7] Watabe T, Ikeda H, Nagamori S, Wiriyasermkul P, Tanaka Y, Naka S (2017). ^18^F-FBPA as a tumor-specific probe of L-type amino acid transporter 1 (LAT1): a comparison study with ^18^F-FDG and 11C-methionine PET. Eur J Nucl Med Mol Imaging.

[8] Bao Q, Newport D, Chen M, Stout DB, Chatziioannou AF (2009). Performance evaluation of the inveon dedicated PET preclinical tomograph based on the NEMA NU-4 standards. J Nucl Med.

[9] Hanaoka K, Watabe T, Naka S, Kanai Y, Ikeda H, Horitsugi G (2014). FBPA PET in boron neutron capture therapy for cancer: prediction of (10)B concentration in the tumor and normal tissue in a rat xenograft model. EJNMMI Res.

[10] Monti Hughes A, Pozzi E, Thorp SI, Curotto P, Medina VA, Martinel Lamas DJ (2015). Histamine reduces boron neutron capture therapy-induced mucositis in an oral precancer model. Oral Dis.

[11] Watabe T, Kanai Y, Ikeda H, Horitsugi G, Matsunaga K, Kato H (2017). Quantitative evaluation of oxygen metabolism in the intratumoral hypoxia: ^18^F-fluoromisonidazole and ^15^O-labelled gases inhalation PET. EJNMMI Res.

[12] Koyasu S, Tsuji Y, Harada H, Nakamoto Y, Nobashi T, Kimura H (2016). Evaluation of tumor-associated stroma and its relationship with tumor hypoxia using dynamic contrast-enhanced CT and ^(18)^F misonidazole PET in murine tumor models. Radiology.

[13] Cruickshank GS, Ngoga D, Detta A (2009). A cancer research UK pharmacokinetic study of BPA-mannitol in patients with high grade glioma to optimise uptake parameters for clinical trials of BNCT. Appl Radiat Isot.

[14] Heikkinen S, Savolainen S, Melkko P (2011). In vitro studies on stability of l-*p*-boronophenylalanine-fructose complex (BPA-F). J Radiat Res.

[15] Watanabe T, Hattori Y, Ohta Y, Ishimura M, Nakagawa Y, Sanada Y (2016). Comparison of the pharmacokinetics between l-BPA and l-FBPA using the same administration dose and protocol: a validation study for the theranostic approach using [^18^F]-l-FBPA positron emission tomography in boron neutron capture therapy. BMC Cancer.

