# Supplementary material for: Practical calculation method to estimate the absolute boron concentration in tissues using 18F-FBPA PET
Source: Ann Nucl Med. 2017 Apr 24;31(6):481–5. doi: 10.1007/s12149-017-1172-5 (PMC5486508; doi:10.1007/s12149-017-1172-5)
Supplement: Supplementary file 1 — Supplementary material 1 (DOC 1606 kb) [file 12149_2017_1172_MOESM1_ESM.doc]

**Supplemental Figures**

**
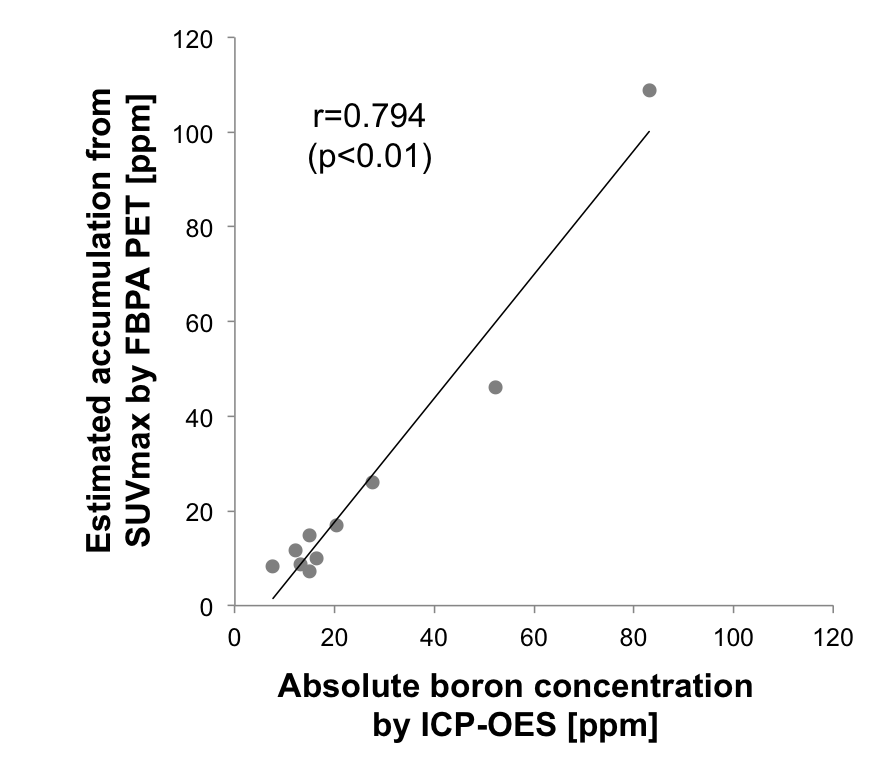
**


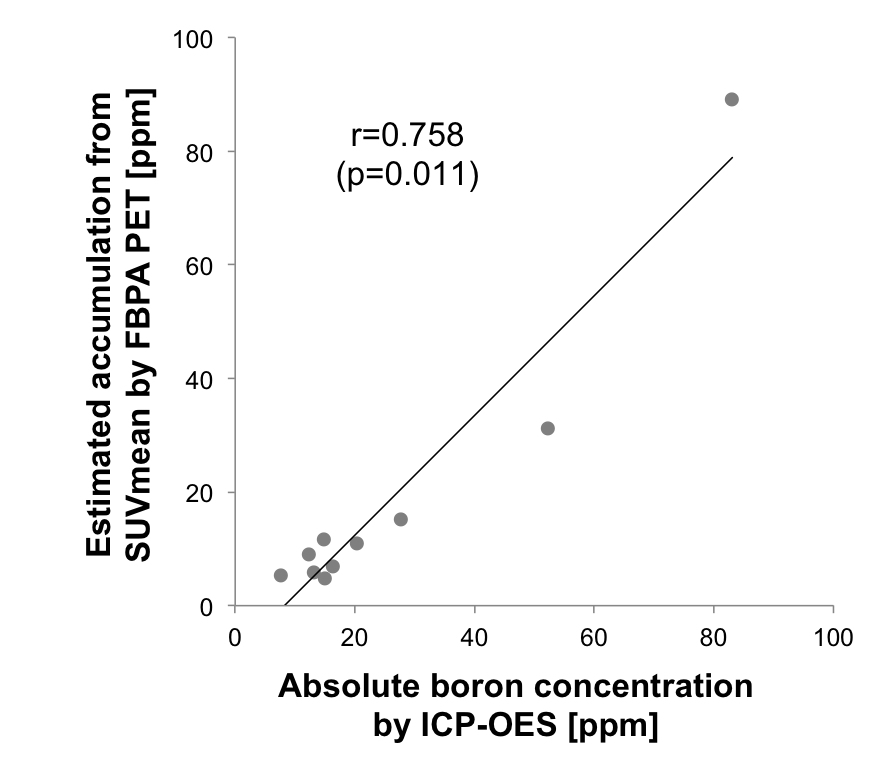


Supplemental Fig. 1. Correlations between the absolute boron concentration and the estimated accumulation from ^18^F-FBPA uptake (correlation analysis by Spearman’s test).


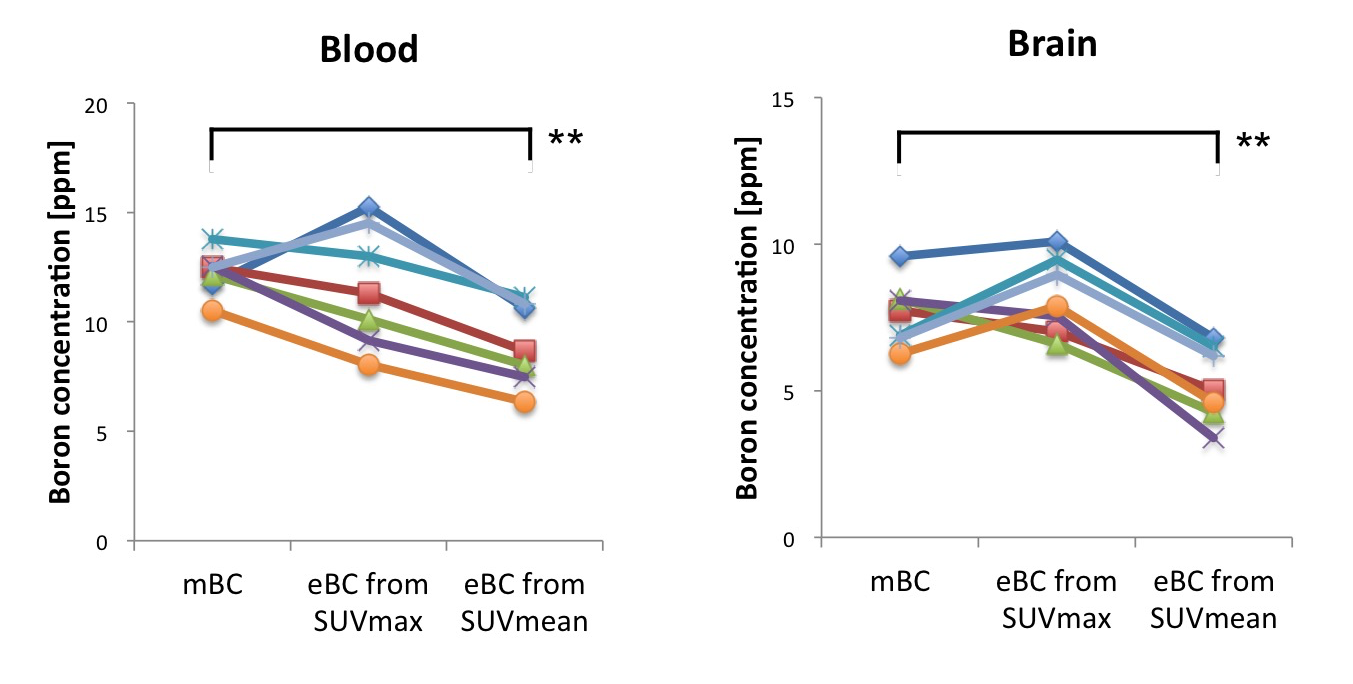


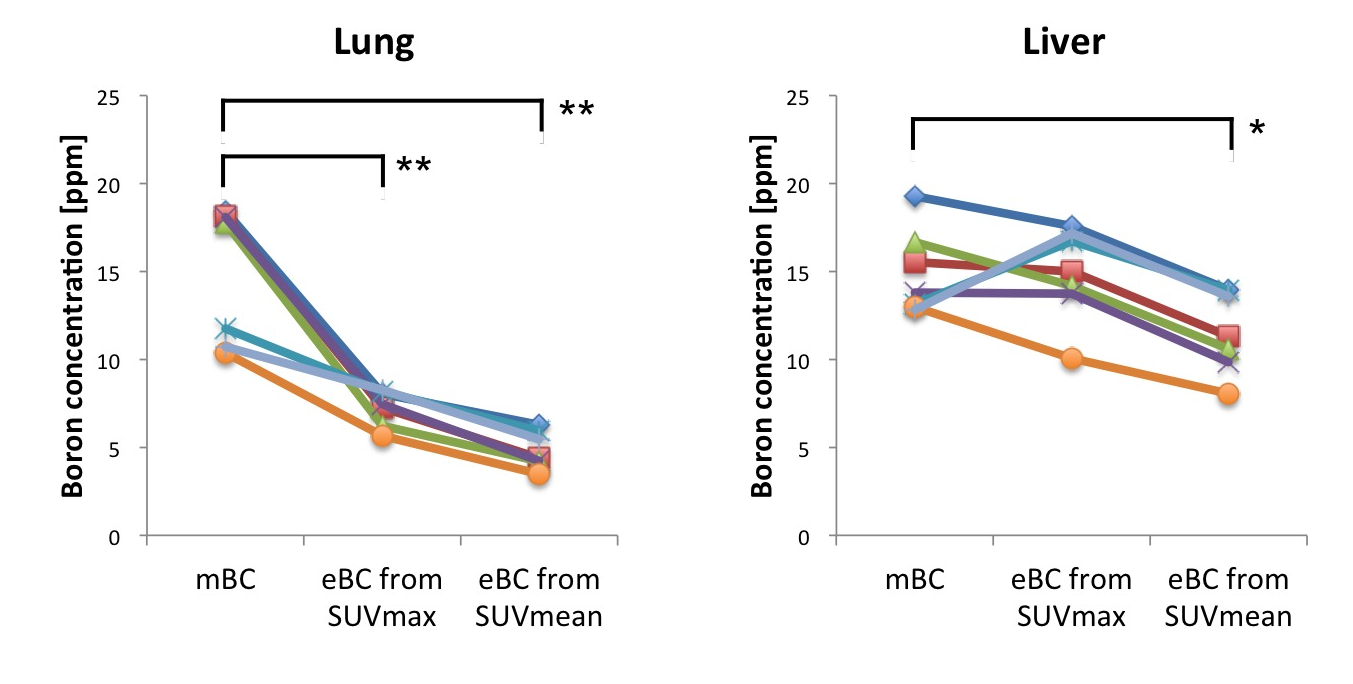


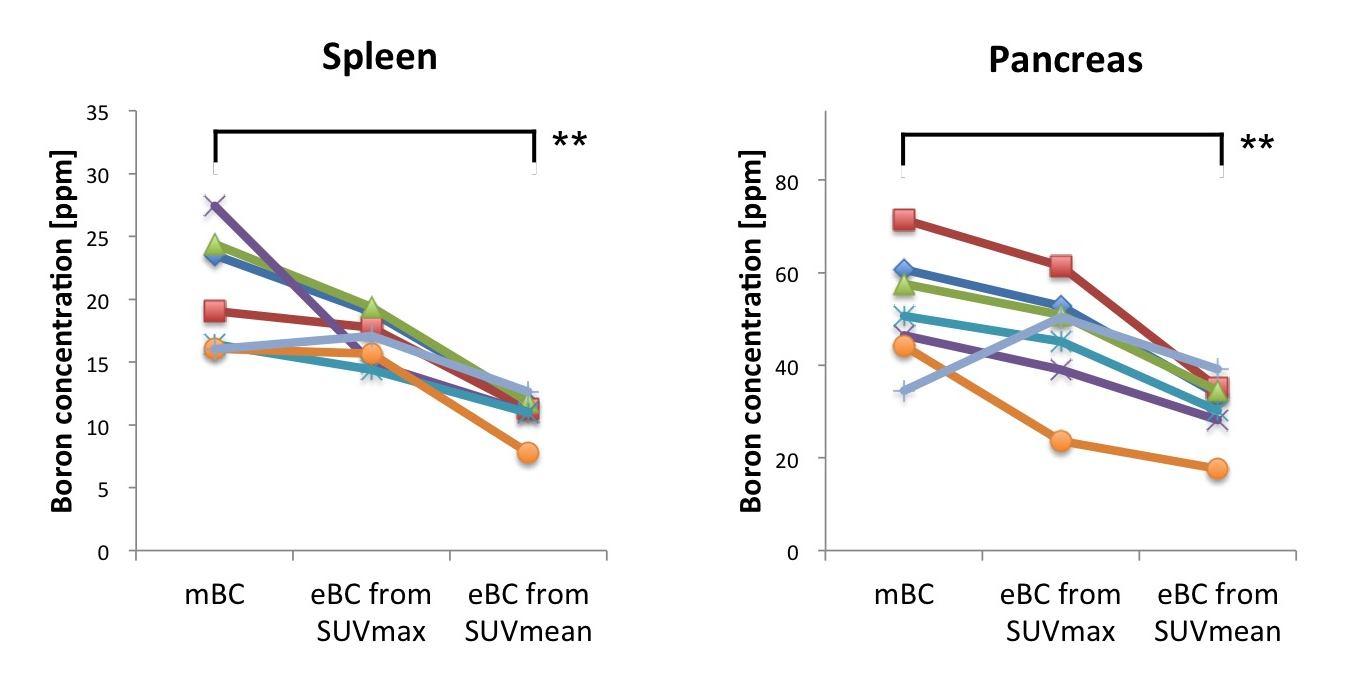


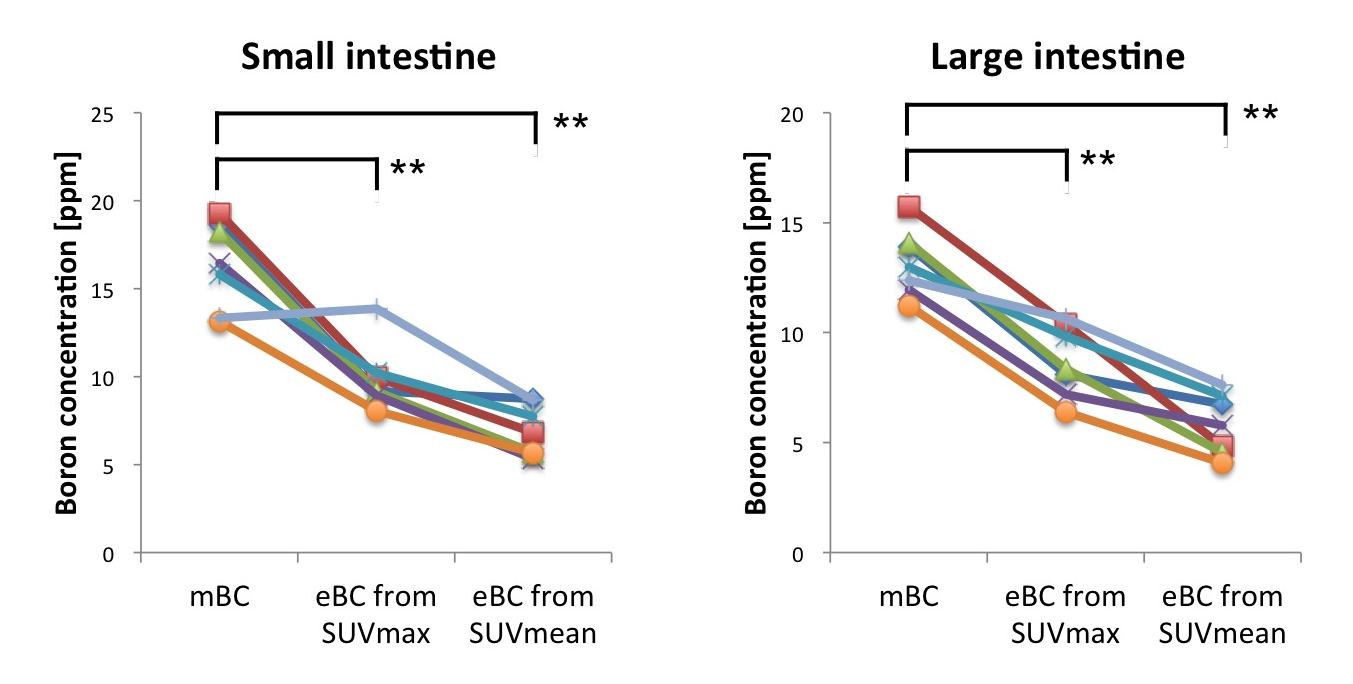


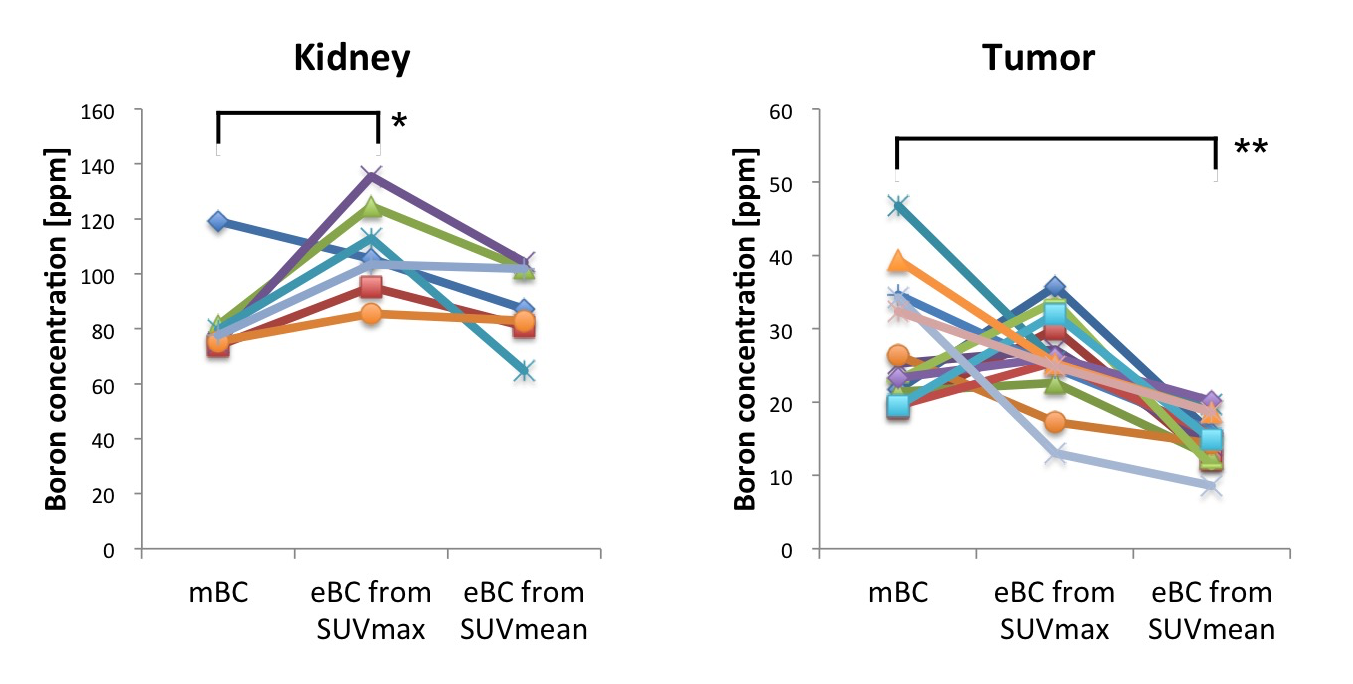


Supplemental Fig. 2. Comparison of the boron concentrations (ppm) between mBC and eBC (calculated from the SUV_max_ or SUV_mean_) (**: p < 0.01 and *: p < 0.05 by paired t-test).
